# Availability and Price of Low-Sodium Condiments and Instant Noodles in the Bangkok Metropolitan Region

**DOI:** 10.5888/pcd20.220218

**Published:** 2023-03-30

**Authors:** Chanatip Chailek, Phanthanee Thitichai, Hirunwut Praekunatham, Pantila Taweewigyakarn, Thanawadee Chantian

**Affiliations:** 1 Field Epidemiology Training Program, Ministry of Public Health, Nonthaburi, Thailand; 2 Division of Epidemiology, Ministry of Public Health, Nonthaburi, Thailand

## Abstract

**Introduction:**

Excess sodium consumption can cause hypertension. One component of Thailand’s 5-part strategy to reduce sodium intake is reform of the food environment to increase access to low-sodium foods. Our research aimed to describe the availability and price of low-sodium food products in retail stores in the Bangkok Metropolitan Region.

**Methods:**

In June and July 2021, we used multistage cluster sampling to conduct a cross-sectional study of the availability of low-sodium foods. Availability referred to a retail store offering at least 1 version of low-sodium condiment or instant noodles. We applied the Thai Healthier Choice criteria and World Health Organization (WHO) global benchmark as the low-sodium criteria for these products. We surveyed 248 retail stores in 30 communities in 6 districts in the Bangkok Metropolitan Region. We observed store shelf availability and price by using a survey form and used the Fisher exact test and independent *t* test to compare availability and price by sodium content and store size.

**Results:**

All subcategories of low-sodium condiments, except black soy sauce in small stores, were less available than regular-sodium condiments. The proportional difference ranged from 11.3% to 90.6% (*P* < .001). We found no difference in the 4 condiment subcategories, including fish sauce, thin soy sauce, seasoning sauce, and oyster sauce in large stores. Low-sodium versions of instant noodles were unavailable in either large or small stores. The price of low-sodium condiments was 2 to 3 times higher than that of regular-sodium condiments (*P* < .05).

**Conclusion:**

Low-sodium food options are not generally available in the Bangkok Metropolitan Region, and access to them is inequitable because of pricing. Instant noodles, a popular food, were unavailable in low-sodium versions. Their reformulation should be promoted. Government subsidies of the price of commonly used low-sodium condiments could increase their use and reduce sodium consumption overall.

**Summary**

**What is already known on this topic?**

In 2021, Thai people consumed 1.8 times as much sodium as the World Health Organization recommended. At least 2 factors contributed to the high sodium intake: a preference for seasoning food with condiments and consumption of instant noodles.

**What is added by this report?**

Low-sodium condiments were less available than regular products, particularly in convenience and traditional stores. The price of low-sodium products was higher than that of regular-sodium versions. No low-sodium instant noodles were available.

**What are the implications for public health practice?**

Collaboration across all sectors of the food supply chain is needed to increase the availability of low-sodium instant noodles and decrease the price of low-sodium condiments to improve equitable access and reduce sodium intake in Thailand.

## Introduction

In 2014, hypertension, the leading risk factor for cardiovascular disease (CVD), was responsible for more than 50,000 deaths among Thai people (1). In 2020, the prevalence of hypertension in Thailand was 25.4% (2). To prevent CVDs, which account for about 25% of deaths from noncommunicable disease (NCD), the World Health Organization (WHO) recommends that sodium intake for adults not exceed 2,000 mg/d and that all member countries reduce sodium intake by 30% by 2025 (3,4).

The mean sodium intake among Thai adults was 3,636 mg per day in 2020, which was 1.8 times what WHO recommended (5). Two main factors contributed to this high sodium intake: the preference for seasoning food with condiments and the consumption of instant noodles (6–8). Sixty-nine percent of Thai people use fish sauce or soy sauce, which contain 6,400.0 to 9,466.7 mg of sodium per 100 ml of sauce, to season food (6,7). Other condiments, including salt, shrimp paste, and oyster sauce, are also typically used in the household (8). Additionally, Thai people consume more than 3 billion servings each year, or about 50 servings per capita, of instant noodles, which contain 1,818.2 to 3,636.4 mg of sodium per 100 grams of noodles (9,10).

The Department of Disease Control of the Ministry of Public Health of Thailand adopted its Salt and Sodium Reduction Strategy (SALTS, 2016–2025) to meet WHO’s global NCD goal. SALTS is a 5-part strategy that stands for stakeholder network, awareness, legislation and environmental reform, technology and innovation, and surveillance, monitoring, and evaluation. The third element of that strategy is to encourage the reformulation and development of low-sodium products and increase access to them (9). If low-sodium products were made more accessible and available through reduced pricing, people might consume less sodium, which could reduce the prevalence of hypertension (11).

To support this strategy, Thailand needed evidence-based statistics on the availability and cost of low-sodium products. Therefore, our study aimed to describe the availability and price of low-sodium and regular-sodium condiments and instant noodles offered in both large and small retail stores in metropolitan Bangkok, the capital city, which has a population of approximately 12 million people (12).

## Methods

### Study design and study site

We conducted a cross-sectional study from June 28 through July 4, 2021, on the availability and price of condiments and instant noodles in retail stores in Bangkok and Nonthaburi, the 2 provinces that make up the metropolitan area of Bangkok.

### Operational definitions

Product availability was defined as the percentage of stores selling at least 1 version of low-sodium or regular-sodium product. The unit of analysis was a retail store, defined as a place selling small amounts of products directly to end consumers. Stores were categorized into 2 types by using the number of cashiers as a proxy for sale area. In Thailand, a hypermarket typically has a sale area larger than 1,000 m^2^. A supermarket has a sale area larger than 400 m^2^, and a convenience store has a sale area larger than 40 m^2^. A large store (hypermarket or supermarket) had 5 or more cashiers. A small store (convenience store or traditional store) had fewer than 5 cashiers. We defined a traditional store as a small retail store owned by local people (13).

Condiments were defined as products used to add flavor to food and were classified into 11 subcategories: fish sauce, thin soy sauce, seasoning sauce, black soy sauce, oyster sauce, chili sauce, tomato sauce, chicken dipping sauce, seafood dipping sauce, jaew spicy dipping sauce, and sukiyaki sauce (14). To identify low-sodium condiments, we used a sodium threshold of the Healthier Choice logo criteria stipulated by the Institute of Nutrition at Mahidol University and the Thai Food and Drug Administration (15). These criteria covered a wide range of condiments with a threshold that depended on each subcategory. For example, the Healthier Choice fish sauce contained 6,000 mg of sodium or less per 100 ml; thin soy sauce, 5,000 mg of sodium or less per 100 ml; and oyster sauce, 2,000 mg of sodium or less per 100 g.

Instant noodles were defined as individually packaged products consisting of partially cooked noodles, various seasonings, and flavored oil (16). Instant noodles were characterized by the type of noodles (egg noodles, rice stick noodles, or rice vermicelli), recipes (with and without soup), and packaging (bag or cup). Low-sodium instant noodles were identified by using WHO’s global benchmark of 770 mg or less of sodium per 100 g of food product (17). Regular-sodium condiments or instant noodles were products with sodium above the Thai Healthier Choice criteria or WHO’s low-sodium benchmark.

### Sample size

The sample size for product availability was calculated by using the proportion formula for an infinite population (18). We assumed the proportion of low-sodium instant noodles to be 76.9% as in a literature review with a 95% confidence level, a precision of 10%, and a design effect of 2 (19). The calculated sample size was 137 stores. The final sample size, yielded from the 30-cluster sampling method, was considered adequate.

We used 2 independent means to calculate the sample size for product price (20). Our assumptions were based on a literature review and the foreign exchange rate in June 2021 (19). We calculated a 95% confidence level and a power of 80%. The calculated sample size was 4 products in both low-sodium and regular-sodium product groups.

### Sampling

We used a multistage random sampling method for selecting retail stores from 30 clusters in 4 districts of Bangkok Province (Pathum Wan, Cha Tu Chack, Bang Khae, and Nong Chock) and 2 districts in Nonthaburi Province (Mueang and Sai Noi). These districts were chosen by their location to represent urban and rural areas of each province. In the first sampling stage, a community was defined as an area where groups of people lived together as established under local government regulation. Specific communities were selected from a government list as the cluster with probability proportional to the size of the population in each district. Because no list of all retail stores was available, we assumed that the population would represent the number of stores in the district. In the second stage, all large stores were selected as data collection stores. To identify small stores in the absence of a list, we walked through each community, starting at the entrance on the main road, and selected the first 7 small stores.

### Data collection

Data collectors were trained officers from the Division of Epidemiology of the Department of Disease Control, Ministry of Public Health. These officers observed products on shelves and recorded data on a survey form adapted from the Nutrition Environment Measures Survey in Stores (21). We pilot-tested the form in 7 large and 3 small stores in the 6 districts that our study area comprised to identify all low-sodium products and the 3 most available regular-sodium products. Variables of interest included the store’s name, type, location, and number of cashiers and product name, category (condiments or instant noodles) and subcategory, food serial number, size (net weight or volume), and price. When various sizes of products were available, the largest size was recorded.

### Data analysis

In each subcategory of condiments and instant noodles, we measured the proportional difference to compare availability of low-sodium and regular-sodium products in the same type of store. We the χ^2^ test or Fisher exact test to analyze product availability between large and small stores. We measured the variety of low-sodium products as a mean number of versions per store, which was the sum of the number of low-sodium versions in each store divided by the number of total stores. This mean was compared among types of stores by using an independent *t* test.

The mean price of an individual product was standardized by volume or weight. The mean price of a product was the sum of the mean price of the individual product divided by the total number of products in each subcategory. We used an independent *t* test to compare the average price between regular and low-sodium products. Significance was defined as *P* < .05. We used R Studio Version 1.2.1335 (Posit Software, formerly R Studio) to analyze data.

### Ethical considerations

We informed retailers about our study’s purpose and methods through an official letter from the Division of Epidemiology and began surveying stores after receiving verbal permission. The institutional review board’s approval was not applicable because this study did not involve research on humans.

## Results

### Characteristics of retail stores

Our study comprised a total of 248 retail stores, with 139 stores in Bangkok and 109 in Nonthaburi. Of these, 17 were hypermarkets (6.9%), 19 supermarkets (7.7%), 114 convenience stores (46.0%), and 98 traditional stores (39.5%). Both large and convenience stores were leading commercial chain stores in Thailand (Table 1).

**Table 1:** Retail Stores (N = 248) by Type^a^, Study of Availability of Low-Sodium Condiments and Instant Noodles, Bangkok and Nonthaburi Provinces, Bangkok Metropolitan Region, Thailand, 2021

Province/district	Hypermarket	Supermarket	Convenience store	Traditional store	Total
**Bangkok, n = 139**					
Pathum Wan	2 (0.8)	6 (2.4)	7 (2.8)	1 (0.4)	16 (6.5)
Cha Tu Chack	2 (0.8)	5 (2.0)	18 (7.3)	11 (4.4)	36 (14.5)
Bang Khae	3 (1.2)	4 (1.6)	18 (7.3)	24 (9.7)	49 (19.8)
Nong Chock	3 (1.2)	1 (0.4)	13 (5.2)	21 (8.5)	38 (15.3)
**Nonthaburi, n = 109**					
Mueang	6 (2.4)	3 (1.2)	51 (20.6)	34 (13.7)	94 (37.9)
Sai Noi	1 (0.4)	0	7 (2.8)	7 (2.8)	15 (6.0)
Total	17 (6.9)	19 (7.7)	114 (46.0)	98 (39.5)	248 (100.0)

a Hypermarkets (sale area >1,000 m^2^), supermarket (sale area >400 m^2^), convenience store (sale area >40 m^2^), traditional store (a small retail store owned by local people). Values are number (percentage).

### Availability and price of condiments

Low-sodium thin soy sauce was available in 94.4% of large stores, and fish sauce, black soy sauce, and chili sauce were available in 91.7% (Table 2). The availability of low-sodium products was significantly lower than regular-sodium products in 6 subcategories, including seafood dipping sauce (a proportion difference of 44.5%, *P* < .001), tomato sauce (38.9%, *P* < .001), chicken dipping sauce (36.1%, *P* < .001), jaew spicy dipping sauce (33.3%, *P* = .008), sukiyaki sauce (27.8%, *P* = .003), and seasoning sauce (19.4%, *P* = .01).

**Table 2:** Availability of Low-Sodium Condiments and Instant Noodles, by Size of Retail Store (N = 248), Bangkok and Nonthaburi Provinces, Bangkok Metropolitan Region, Thailand, 2021^a^

Category/ subcategory	Large stores^b^ (n = 36)			Small stores^b^ (n = 212)		
	Low-sodium product	Regular-sodium product	*P* value^c^	Low -odium product	Regular-sodium product	*P *value^c^
**Condiment**						
Fish sauce	33 (91.7)	36 (100.0)	.24	17 (8.0)	208 (98.1)	<.001
Thin soy sauce	34 (94.4)	36 (100.0)	.50	10 (4.7)	195 (92.0)	<.001
Seasoning sauce	29 (80.6)	36 (100.0)	.01	42 (19.8)	199 (93.9)	<.001
Black soy sauce^d^	33 (91.7)	NA	NA	145 (68.4)	NA	NA
Oyster sauce	32 (88.9)	36 (100.0)	.12	6 (2.8)	193 (91.0)	<.001
Chili sauce	33 (91.7)	36 (100.0)	.24	5 (2.4)	172 (81.1)	<.001
Tomato sauce	22 (61.1)	36 (100.0)	<.001	1 (0.5)	172 (81.1)	<.001
Chicken dipping sauce	23 (63.9)	36 (100.0)	<.001	2 (0.9)	172 (81.1)	<.001
Seafood dipping sauce	17 (47.2)	33 (91.7)	<.001	8 (3.8)	71 (33.5)	<.001
Jaew spicy dipping sauce	15 (41.7)	27 (75.0)	.008	0 (0.0)	24 (11.3)	<.001
Sukiyaki sauce	25 (69.4)	35 (97.2)	.003	4 (1.9)	196 (92.5)	<.001
**Instant noodles^e^**						
Egg noodles, bag						
Soup recipe	NA	36 (100.0)	<.001	NA	165 (77.8)	<.001
Without soup recipe	NA	36 (100.0)	<.001	NA	148 (69.8)	<.001
Egg noodles, cup						
Soup recipe	NA	36 (100.0)	<.001	NA	138 (65.1)	<.001
Without soup recipe	NA	36 (100.0)	<.001	NA	16 (7.5)	<.001
Rice stick noodles, cup	NA	31 (86.1)	<.001	NA	43 (20.3)	<.001
Rice vermicelli, cup	NA	14 (38.9)	<.001	NA	53 (25.0)	<.001

Abbreviation: NA, not applicable.

a Values are number (percentage) unless otherwise indicated.

b Number of cashiers was used as a proxy for store size. A large store (hypermarket or supermarket) had 5 or more cashiers. A small store (convenience store or traditional store) had fewer than 5 cashiers.

c *P* values calculated by using χ^2 ^test or Fisher exact test.

d Black soy sauce was available only in low-sodium versions.

e Instant noodles were not available in a low-sodium version.

Low-sodium black soy sauce was available in 68.4% of small stores, followed by seasoning sauce (19.8%) and fish sauce (8.0%). Low-sodium products, except for black soy sauce, which had no regular-sodium versions, were significantly less available than regular-sodium products in all subcategories with proportional differences ranging from 11.3% to 90.6% (*P* < .001). Small stores had less availability of low-sodium condiments in all subcategories than large stores, including commonly used household sauces: fish sauce (8.0% vs 91.7%, *P* < .001), thin soy sauce (4.7% vs 94.4%, *P* < .001), and oyster sauce (2.8% vs 88.9%, *P* < .001). Regular-sodium fish sauce, thin soy sauce, seasoning sauce, and oyster sauce in both size stores were comparably available (Table 2).

The mean number of low-sodium versions for each subcategory of all condiments was significantly lower in small stores (*P* < .001), except black soy sauce. On average, large stores had 3.4 (SD, 1.2) different low-sodium versions of thin soy sauce, compared with an average of 0.1 (0.3) low-sodium version in small stores. In small stores, most condiments did not have any low-sodium versions, apart from seasoning sauce (mean, 0.2; SD, 0.4), thin soy sauce (mean, 0.1; SD, 0.3), and fish sauce (mean, 0.1; SD, 0.3) (Figure).

**Figure:**
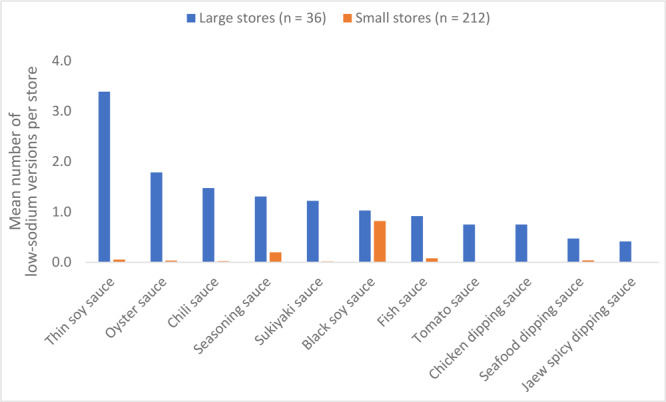
Number of versions of low-sodium condiments available per store by size of store, Bangkok and Nonthaburi Provinces, Bangkok Metropolitan Region, Thailand, 2021. The number of cashiers was used as a proxy for store size. A large store (hypermarket or supermarket) had 5 or more cashiers. A small store (convenience store or traditional store) had fewer than 5 cashiers. Differences were calculated by using independent *t* test.

**Table:** 

Condiment	Large stores (n = 36)	Small stores (n = 212)	*P *value
Thin soy sauce	3.4 (1.2)	0.1 (0.3)	<.001
Oyster sauce	1.8 (0.9)	0.0 (0.2)	<.001
Chili sauce	1.5 (0.7)	0.0 (0.2)	<.001
Seasoning sauce	1.3 (0.8)	0.2 (0.4)	<.001
Sukiyaki sauce	1.2 (0.9)	0.0 (0.1)	<.001
Black soy sauce	1.0 (0.4)	0.8 (0.7)	.067
Fish sauce	0.9 (0.3)	0.1 (0.3)	<.001
Tomato sauce	0.8 (0.7)	0.0 (0.1)	<.001
Chicken dipping sauce	0.8 (0.6)	0.0 (0.1)	<.001
Seafood dipping sauce	0.5 (0.5)	0.0 (0.2)	<.001
Jaew spicy dipping sauce	0.4 (0.5)	0.0 (0.0)	<.001

The most expensive low-sodium condiments were jaew spicy dipping sauce (52.7 Baht/100 g), followed by sukiyaki sauce (24.1 Baht/100 g) and thin soy sauce (19.6 Baht/100 ml). Low-sodium condiments were more expensive than regular-sodium condiments, with significant differences in jaew spicy dipping sauce (*P* = .004), thin soy sauce (*P* = .04), tomato sauce (*P* = .002), seasoning sauce (*P* = .001), and fish sauce (*P* = .01). Furthermore, low-sodium jaew spicy dipping sauce had the greatest relative price (3.7 times as expensive as regular-sodium version), followed by thin soy sauce (3.0 times) and seasoning sauce (2.5 times) (Table 3).

**Table 3:** Price Comparison, Low-Sodium and Regular-Sodium Condiments Available in Retail Stores (N = 248), Bangkok and Nonthaburi Provinces, Bangkok Metropolitan Region, Thailand, 2021

Category/subcategory	Price, mean (SD) [n], Baht/100 volume or weight^a^		
	Low-sodium product	Regular-sodium product	*P* value^b^
Fish sauce	11.73 (NA^c^) [1]	5.18 (1.12) [3]	.01
Thin soy sauce	19.61 (8.34) [5]	6.61 (1.52) [3]	.04
Seasoning sauce	13.38 (0.71) [2]	5.35 (0.42) [3]	.001
Black soy sauce^d^	4.94 (2.20) [2]	NA	NA
Oyster sauce	10.38 (11.76) [5]	7.86 (2.16) [2]	.79
Chili sauce	16.32 (11.81) [4]	9.01 (2.04) [4]	.27
Tomato sauce	18.49 (1.23) [2]	9.60 (0.74) [3]	.002
Chicken dipping sauce	13.14 (5.08) [2]	8.78 (1.86) [2]	.37
Seafood dipping sauce	14.21 (NA^c^) [1]	18.73 (4.52) [2]	.39
Jaew spicy dipping sauce	52.67 (NA^c^) [1]	14.25 (4.20) [3]	.004
Sukiyaki sauce	24.12 (19.16) [4]	14.42 (2.53) [2]	.54

Abbreviation: NA, not applicable.

a Values are mean (SD) [number] in Baht per 100 volume or weight. The units for volume or weight were millimeter or gram.

b *P* value calculated by using independent *t* test.

c SD could not be calculated because only 1 low-sodium version was available.

d Black soy sauce is available only in low-sodium versions.

### Availability and price of instant noodles

No instant noodles were available in a low-sodium version, and none met WHO’s standard for low sodium content (Table 2).

## Discussion

In small stores, all subcategories of low-sodium condiments were substantially less available than regular-sodium condiments with a proportional difference up to 90%. In large stores, 6 subcategories of low-sodium condiments were substantially less available: 35% of large stores did not offer 6 subcategories of low-sodium condiments. Low-sodium condiments were more expensive than regular-sodium condiments. Although inexpensive instant noodles in all 3 subcategories were sold in both small and large stores, no instant noodles that met WHO’s low-sodium global benchmark were available. In our study area of the metropolitan capital city of Bangkok, availability and affordability of low-sodium condiments and instant noodles were limited, highlighting the inequity of access to healthy food.

Less than 10% of small stores had low-sodium versions of commonly used household condiments (fish sauce, soy sauce, and oyster sauce). As a 2015 study in Guam found, small stores sold fewer low-sodium items, including soy sauce, than large stores (19). In contrast, more than half of large stores offered low-sodium options in 9 of the 11 condiment subcategories we studied, demonstrating a product assortment strategy to fulfill consumer needs and expectations (22,23). Hypermarkets and supermarkets generally focus on middle- to upper-class consumers, whereas all consumers can easily access convenience and traditional stores (13). These market differences could create unequal access to low-sodium options, especially among low-income consumers. However, the product assortment strategy in small stores is usually challenging because of limited product display area.

The only condiment that had no regular-sodium equivalent was black soy sauce. We found that all products of this condiment met the Thai Healthier Choice criteria. Black soy sauce is used in approximately one-third of Thai households for stir-frying dishes such as fried rice and fried noodles or as a dipping sauce (8,24). The Healthier Choice criterion for black soy sauce and thin soy sauce was the same (sodium <5,000 mg/100 ml) although sweetness was the most prominent characteristic of black soy sauce (15).

Most low-sodium condiments were more expensive than regular ones. This finding was in line with the 2015 Guam study (19). Although fish sauce and thin soy sauce were commonly used, their prices were 2 to 3 times higher than regular-sodium products. One possible explanation could be that potassium chloride, a salt substitute used in reformulation of those condiments, costs 1.1 to 14.6 times as much as sodium chloride (25). Thus, low-income consumers might not be able to afford low-sodium options, emphasizing the inequity of access to healthy food (26).

Instant noodles are a common dish in Thailand (10). The proportion of people who eat instant foods 1 to 2 days per week increased from 47.8% in 2013 to 59.3% in 2017 (6). However, although over 20 instant noodles passed the Healthier Choice criterion of less than 1,000 mg per 50 g of noodles, this was still 2.6 times that of WHO’s benchmark (<770 mg sodium/100 g of food product) (17,27). Thai consumers might be misled by the Healthier Choice logo and consume these products that contain more salt than anticipated.

Our study had several limitations. First, because of cluster sampling, our findings could not be statistically inferred to the target population. However, we stratified districts in the Bangkok and Nonthaburi regions by zoning, and our sample still reflects the diversity of stores in the Bangkok Metropolitan Region. Second, 2 products of black soy sauce were not labeled with complete nutritional information. We could not identify whether they were low-sodium or regular-sodium products. Additionally, other condiments that are sources of sodium in Thailand, such as shrimp paste, seasoning powder, and fermented fish sauce, were not included in our study because there were no established Healthier Choice criteria for these products. Third, product prices might vary over time. However, we completed the survey in 1 week, and the price comparison was unlikely to be influenced during this time. Finally, in terms of access, this study focused only on availability and price of low-sodium products; customer acceptability and willingness to pay should be explored in future studies.

## Conclusions

From a survey of 248 retail stores in 30 communities in 6 districts in the metropolitan area of Bangkok, our study found that the availability of low-sodium condiments was significantly less than that of regular-sodium products, particularly in small stores. The price of the low-sodium products was higher than that of regular condiments, demonstrating inequitable access to healthy foods. Because no low-sodium version of instant noodles was available, this popular food continues to contribute to a high sodium diet. Reformulation of instant noodles could be promoted, and convenience stores could receive incentives and subsidies to increase availability and reduce the price of low-sodium condiments.

### Recommendations

The Thai Food and Drug Administration and the Ministry of Industry should promote the reformulation of instant noodles to meet WHO’s global benchmark to increase the availability of a low-sodium version of this popular food product. In collaboration with the Ministry of Commerce, the Ministry of Public Health should create incentives or require small stores to offer low-sodium condiments (28). Despite their limited space, chain convenience stores might manage stock better than traditional stores because they have distribution centers and higher technology. Public health campaigns encouraging people to replace current condiments with low-sodium options might raise product demand in the community. Furthermore, interventions, such as subsidies or discounts, could be implemented to reduce the cost of low-sodium condiments, particularly those most commonly used by households. This could result in more equitable access to healthy foods.
